# Towards TB elimination in Malawi: a 5-year analysis of key indicators for TB control using surveillance data

**DOI:** 10.5588/ijtldopen.25.0316

**Published:** 2025-10-10

**Authors:** H.H. Twabi, T.C. Msosa, M. Mukoka, I. Mwaluka, B. Girma, T. Sikwese, J. Mpunga, T. Mwenyenkulu, D. Chimatiro, K. Mbendera, J. Simbeye, M. Nliwasa

**Affiliations:** ^1^ Kamuzu University of Health Sciences, Blantyre, Malawi;; ^2^ Institute of Life Course and Medical Sciences, University of Liverpool, Liverpool, UK;; ^3^ Malawi-Liverpool-Wellcome Programme, Blantyre, Malawi;; ^4^ Department of Global Health, Amsterdam UMC, University of Amsterdam, Amsterdam, the Netherlands;; ^5^ Amsterdam Institute for Global Health and Development, Amsterdam, the Netherlands;; ^6^ Department of Infectious Disease Epidemiology and International Health, London School of Hygiene and Tropical Medicine, London, UK;; ^7^ National Tuberculosis and Leprosy Elimination Programme, Ministry of Health, Lilongwe, Malawi;; ^8^ University of Malawi, Zomba, Malawi.

**Keywords:** tuberculosis, epidemiology, case notifications, trends, Malawi

## Abstract

**BACKGROUND:**

Malawi’s TB Control Programme emphasises data-driven approaches for monitoring TB control efforts, but programmatic indicators have never been systematically evaluated. This study evaluates the performance of Malawi’s TB Control Programme, providing insights into national trends, geographical distributions, and programmatic gaps in TB care.

**METHODS:**

Aggregate TB data collected through Malawi’s District Health Information System from 2018 to 2022 were analysed cross-sectionally. We analysed trends in TB incidence and case notification rates (CNRs), calculated performance indicators, and assessed district-level variations using time-series plots and statistical comparisons. Population estimates were derived from the 2018 census and adjusted for annual growth.

**FINDINGS:**

Malawi reported 18,025 new persons with TB in 2022. From 2005 to 2018, TB incidence and CNRs declined by 68.6% and 54.5%, respectively. The highest CNRs were recorded among men aged 35–64 years. Treatment success rates improved overall, reaching 89.2% in 2022, though disparities persisted for HIV-positive patients and those treated at tertiary facilities.

**CONCLUSION:**

Challenges remain in Malawi’s TB control efforts, particularly in addressing case detection gaps in high-burden districts and improving outcomes for vulnerable populations. Strengthening active case finding, enhancing diagnostic capacity, and addressing socio-economic determinants of health are essential for sustaining progress and achieving END-TB Strategy goals.

TB is the leading cause of infectious mortality worldwide, with an estimated 1.25 million deaths (95% uncertainty interval [UI]: 1.13–1.37 million) occurring in 2023, including 161,000 deaths among people living with HIV (PLHIV).^1^ The burden of TB is more pronounced in less economically developed settings such as Africa, which alone accounted for 23% of new TB cases and 31% of TB-related deaths globally in 2023.^1^ The TB epidemic in sub-Saharan Africa (SSA) has primarily been driven by widespread poverty and the HIV/AIDS pandemic. Malawi is one of the countries with a high burden of HIV-associated TB in the world.^1^ The country registered an estimated 25,000 (95% UI: 12,000–44,000) new cases and 4,800 deaths in 2023.^2^ These numbers may be underestimations owing to the persistent challenges in health care access and optimal TB diagnostics.^3–5^ Expanded access to antiretroviral therapy (ART) has reduced the overall burden of HIV, resulting in declining trends in TB case notifications in the SSA region, including in Malawi.^6^ Despite this, the World Health Organization (WHO) still estimates that the true incidence of TB is higher than the numbers notified through the routine TB registers,^1^ suggesting that some cases of the disease are missed. A study by Khundi et al.^7^ in 2022 that modelled prevalence-to-notification ratios using 2019 data found significant underdiagnosis, particularly in peri-urban informal settlements. Various programmatic and research strategies have been implemented to address this, including active case finding (ACF) as well as novel approaches to facility-based identification of cases.^3,8^ The Malawi National Tuberculosis and Leprosy Elimination Programme (NTLEP) launched the National Tuberculosis and Leprosy Control Strategic Plan (2021–2025) to guide efforts towards eliminating the TB epidemic in Malawi.^9^ The document extensively discusses data collection, reporting, and disease surveillance as crucial components of the strategic plan. In addition to NTLEP’s efforts, several implementing partners – such as Dignitas International and Médecins Sans Frontières – have supported targeted interventions among high-risk populations, including prisoners,^10,11^ children,^12^ and PLHIV.^13,14^ Furthermore, the *Leaving No-One Behind: Transforming Gendered Pathways to Health for TB* (LIGHT) Consortium has developed strategies to close the TB case detection gap, particularly among men,^15^ who continue to bear a higher burden of TB than women.^16–18^ Despite these initiatives, progress towards achieving the strategic plan’s performance indicators has not been formally evaluated since the launch of the 2021–2025 National Tuberculosis and Leprosy Control Strategic Plan. We aim to address the TB surveillance gap in Malawi by integrating routine data sources to describe the trends and spatial distribution of TB in the country.

## METHODS

This is a cross-sectional analysis of data collected during the conduct of the Partnership to Enhance Technical Support for Analytical Capacity and Data Use in East and Southern Africa (PERSuADE) project, a multinational project aimed at strengthening data analysis and utilisation at national and subnational levels to enhance health programme effectiveness and sustainability. Data were obtained centrally from the NTLEP and included data from health facilities from all the 28 districts in Malawi from 2018 to 2022.

### Data collection

Aggregate health facility data on TB are collected routinely using a standardised paper-based reporting tool designed by the NTLEP. The NTLEP conducts periodic trainings for TB officers, as well as supervision visits and TB review meetings to ensure validity and completeness of data entry. Data are collected by the facility TB officers and sent to the Health Management Information Systems officer at the District Health Office, who then enters the data directly onto the electronic District Health Information Systems (DHIS2) platform. Population estimates for the year 2018 were obtained from the 2018 census data that are available through the Malawi National Statistical Office (NSO) website.^19^ Population estimates for the subsequent years up to 2022 were generated using the estimated population growth rate provided by the NSO.

### Definitions of indicators and outcomes

Incidence of TB is defined as the estimated number of new and relapse TB cases arising each year per 100,000 population. All forms of TB are included, including cases in PLHIV. Since TB incidence cannot be directly measured, estimates are obtained by eliciting expert opinion or are derived from measurements of prevalence. These estimates are obtained from the WHO Global Tuberculosis Reports.^1^ TB case notification rates (CNRs) were defined as the number of new and relapse TB cases (including cases with unknown previous TB treatment history) notified during the reporting period (numerator) over the estimated population during the same period (denominator) per 100,000 population. Values for the CNRs from 2005 to 2017 were obtained from the Global TB reports from the corresponding years,^20^ while those for 2018–2022 were calculated from the DHIS2 data. We used case definitions for the various forms of TB outlined in the 2024 National Tuberculosis and Leprosy Guidelines of Malawi.^21^ These include definitions for the TB treatment outcome measures. The TB treatment success rate (TSR) is the percentage of all TB cases registered under a national TB control programme in a given year that successfully completed treatment, with or without bacteriological evidence of success. The WHO’s END-TB strategy aims for at least 90% TSR,^22^ while the National Tuberculosis and Leprosy Control Strategic Plan 2021–2025 has a target of at least 92%.^9^

### Statistical analysis

We reported absolute cases notified for each year and applied time-series plots to visualise yearly variations in TB programme indicators from 2019 to 2022. Subnational notifications were illustrated using spatial maps drawn using opensource shapefiles.^23^ All the analyses were conducted in R statistical computing software (R version 4.3.0 [2023-04-21 – R Core Team (2023), https://www.R-project.org/]).

### Ethical statement

The DHIS2 platform is a routine surveillance platform that collects aggregate facility-level count data and does not include individual-level data. The authors of the article obtained permission to use the data for this analysis from the Malawi Ministry of Health’s NTLEP. This study was conducted in accordance with the ethical standards of the General Data Protection Regulation.

## RESULTS

**Figure 1.**
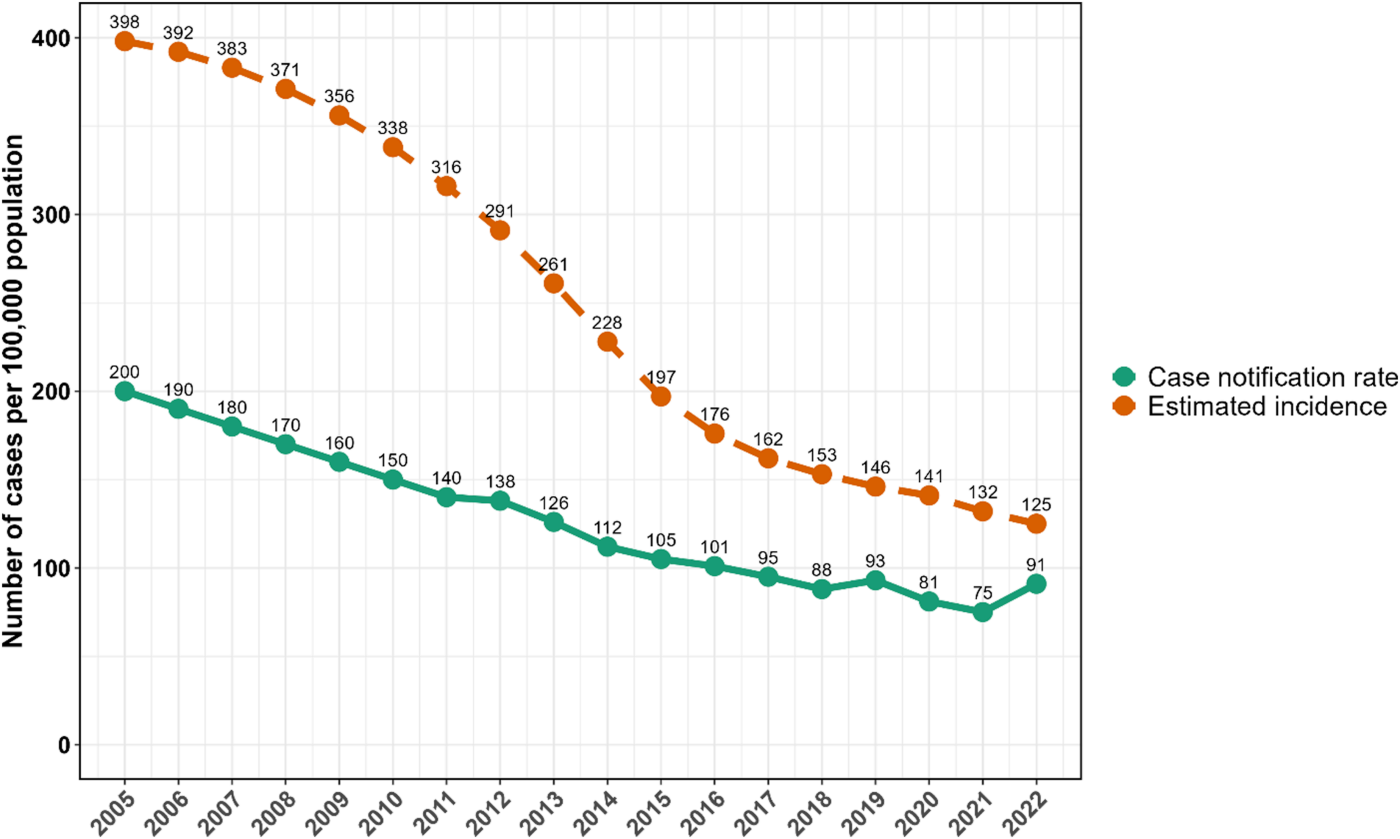
Estimated TB incidence for Malawi (as per WHO Global TB Report estimates) and case notification rates from 2018 to 2022.

Malawi registered a total number of 18,025 new and relapse cases of TB in 2022, a 17.2% increase in cases from 2018 (see Table). Bacteriologically confirmed TB cases peaked at 7,045 cases in 2022, a 22.5% rise compared with 5,750 cases in 2018. Similarly, extra-pulmonary TB cases increased significantly from 5,314 in 2018 to 6,719 in 2022, despite a dip in 2020 and 2021. Figure 1 illustrates the trends in the estimated TB incidence and TB CNRs nationally in Malawi from 2005 to 2022. There is an overall decline in the incidence (by 68.6%) and CNR (by 54.5%) of TB in Malawi over the 18 years of observation. There was an unprecedented drop in the CNRs between 2020 and 2021 (81 and 75 per 100,000, respectively) with a rebound increase in 2022 (91 per 10,000 population). There remains a difference of about 31 cases per 100,000 population between the estimated incident cases of TB and the CNRs by 2022.

**Table.** Summary of TB cases identified in Malawi from 2018 to 2022.

Category of patients	2018	2019	2020	2021	2022
Bacteriologically confirmed TB	5,750	6,157	6,074	6,004	7,045
EPTB	5,314	5,488	4,629	4,682	6,719
Clinically diagnosed TB	3,421	3,950	3,250	2,679	2,981
TB relapse	889	1,317	1,180	1,020	1,280
Total new and relapse cases	15,374	16,912	15,133	14,385	18,025
All TB cases (including old and LTFU)	15,892	17,140	15,380	14,594	18,255

EPTB = extra-pulmonary TB; LTFU = loss to follow-up.

### Geographical distribution of TB cases

**Figure 2.**
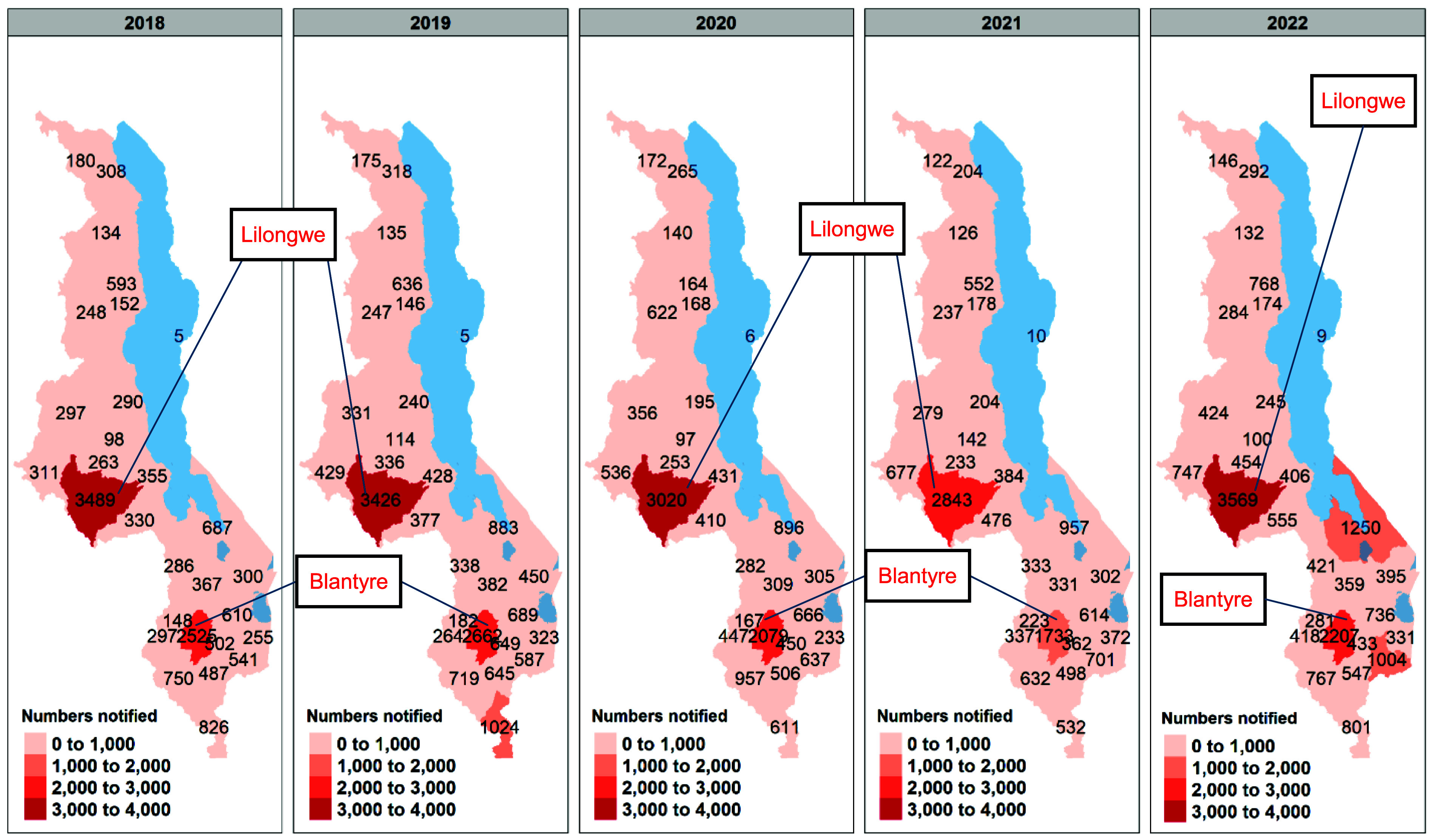
Annual number of all forms of notified TB cases by district highlighting Blantyre and Lilongwe (the two high-burden districts). The figure over the waters of Lake Malawi represents the number of cases identified in Likoma district, an island on Lake Malawi.

There is a lot of variability in TB disease burden by district (see Figure 2). The highest number of cases notified are from Lilongwe (3,532 cases in 2022), followed by Blantyre (2,207 cases in 2022). These districts also fell short of the case detection targets set by the NTLEP (notification-to-target ratio [NTTR] of 0.79 and 0.80 for Lilongwe and Blantyre districts, respectively – Supplementary Data Figure S1). Nsanje district registered an unprecedented high number of cases in 2019 (1,016 cases), as did Mangochi in 2022 (1,225 cases). There has been an increase in the total number of health facilities providing TB services over the years, from 356 in 2018 to 523 in 2022 (Supplementary Data Figure S2). The high-TB-burden districts of Lilongwe and Blantyre have the highest number of TB treatment units at 47 and 43 units, respectively, in 2022.

### TB CNRs by age and gender

**Figure 3.**
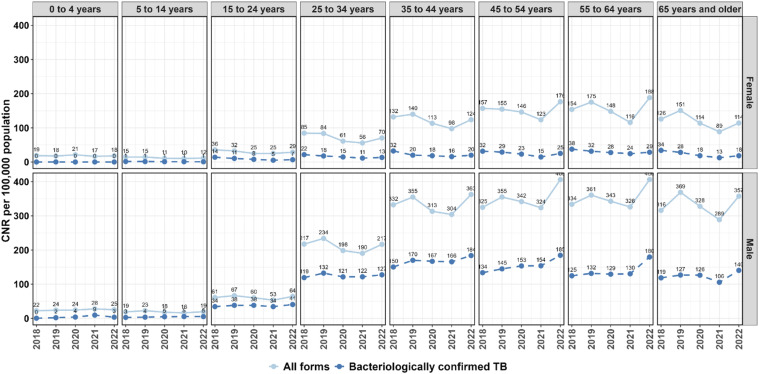
Trends in quarterly TB case notification rates by age and sex from 2018 to 2022. CNR = case notification rate.

Disaggregated yearly trends in TB CNRs per 100,000 are illustrated in Figure 3. The highest CNRs for bacteriologically confirmed TB were registered by men between the ages of 35 and 64 years especially in the year 2022 (CNR of 184, 185, and 180 per 100,000 population for ages 35–44, 45–54, and 55–64 years in 2022, respectively). Men of all age groups had higher TB CNRs than women across the entire period of observation. Men had an increasing trend in CNRs from 2018 to 2022, and women a declining trend within the same period.

### TB notifications by HIV status

**Figure 4.**
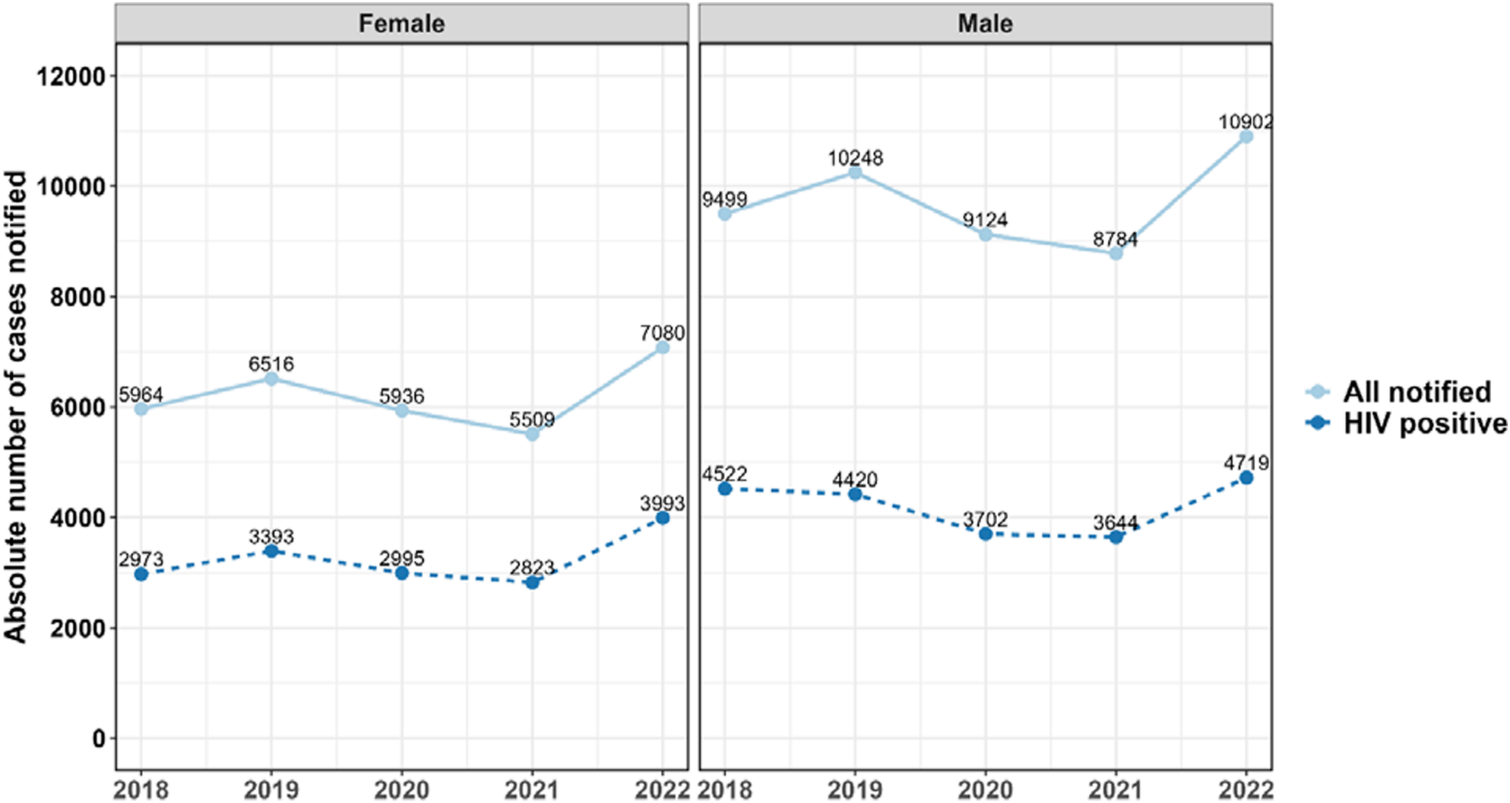
TB case notifications in Malawi by sex and HIV status from 2018 to 2022.

In the year 2022, out of the 18,025 people notified with TB, 8,712 (48.3%) were HIV positive. Of the 8,712 HIV-positive individuals, 8,624 (99%) were on ART, demonstrating excellent HIV/TB service integration. There is a declining trend in the number of HIV-positive people with TB between 2019 and 2021 for both men and women, with a rebound increase observed in 2022 (Figure 4).

### TB TSR

The TSR has been increasing across the observation period, from 85.3% in 2018 to 89.2% in 2022 (Supplementary Data Figure S3). The outcomes for children of all ages are 90% and above in all facility types. For the aggregated adult cohort (both HIV negative and positive), the TSR is high at primary and secondary facility level (88.5% and 92.5%, respectively, in 2022) but remains relatively lower at 83.3% at tertiary care level in 2022. This is more pronounced in the cohort of people who are HIV positive, where the TSR is 80.3% at tertiary care level in 2022.

## DISCUSSION

This is the first study to evaluate the performance of the Malawi TB control programme using routine data. We found a declining incidence and CNR of TB in Malawi, with a focussed epidemic in Lilongwe and Blantyre districts. There remains a gap between the notified cases of TB and the estimated TB incidence in the country.

There has been a steady decline in the CNR for TB in Malawi since 2005, a comparable trend to the estimates of incidence reported by the WHO.^1,24^ However, the rate of decline has slowed down from 2017. Several factors may contribute to this, including challenges in diagnosis and treatment of hard-to-reach populations. Additionally, ongoing socio-economic factors, such as poverty and malnutrition, sustain TB transmission through direct causal association with the disease,^25,26^ as well as impeding access to TB care services.^27–29^ This is further compounded by diminishing funding for TB in a country where TB services have been completely free since 2019.^24^ Further, health system constraints, including human resource shortages and diagnostic capacity limitations, negatively affect TB case finding and reporting.^29^ Another contributor to the inconsistent trends of TB epidemiology in Malawi was the COVID-19 pandemic, which impacted delivery of routine health services, particularly between 2020 and 2022. The global TB incidence rate is estimated to have increased by 3.9% between 2020 and 2022.^24^ In Malawi, the CNR increased by 12.3% over the same period. This reflects disruptions to essential TB services during the COVID-19 pandemic,^30^ with a return to approximate baseline notifications in 2022.

Despite declining trends in case notification, there remains a gap between the CNR and the incidence estimates, which reflects the so-called ‘missing millions’ of undiagnosed and untreated active TB cases who are not captured by routine case identification efforts.^31^ The Malawi NTLEP has instituted some interventions to begin to close this gap. There has been doubling of the number of TB registration sites across the country, thus reducing the distance between the patient and the TB registration facility, a contributor to poor health seeking behaviour.^3^ Another intervention is ACF of TB patients through the use of mobile diagnostic units – mobile vans equipped with diagnostic tools to facilitate TB screening within communities. Evidence to support ACF interventions demonstrates mixed effects,^32–34^ but have potential to reduce the community-level TB prevalence if conducted with ‘sufficient intensity and coverage’.^32^

The provision of TB services is centrally coordinated by the NTLEP. Over the years, the NTLEP has increased the number of health facilities providing TB diagnostic and treatment services nationally. Expansion of facilities reduces the minimum distance to TB services for each individual patient, which has been shown to be associated with up to a 20% increase in the case notifications.^3^ This intervention also reduces the time to diagnosis of each individual person with TB, thus improving treatment outcomes. In addition, the NTLEP and its partners have implemented targeted interventions such as expanding TB preventive therapy for children and PLHIV,^13^ and enhancing case detection efforts in prisons.^10,11^ However, challenges still exist with respect to people’s access to services, with estimates indicating that only 60% of all people with incident TB disease in a year complete treatment.^35^ Continued investment in the availability and accessibility of diagnostic and treatment services is thus imperative to closing the TB care gap.

Lilongwe and Blantyre districts notify the highest number of persons with TB and are the districts where the case detection gap is the largest. These districts contain large urban hubs with high population densities, which facilitates TB transmission.^36^ These districts also face significant challenges related to socio-economic determinants of health, including higher levels of poverty, overcrowded living conditions, and greater proportions of informal settlements, which create barriers to accessing health care services. Moreover, health care systems in these urban areas are overburdened due to high patient volumes, resulting in delays in diagnosis and treatment. Addressing the case detection gap in these districts will require targeted interventions, including the scale-up of ACF through mobile diagnostic units, intensified contact tracing, and expanded screening among key populations. These efforts should be complemented by enhanced community-based TB awareness campaigns and strengthened diagnostic and treatment capacity at health facilities to manage the increased burden of disease. Tailoring these strategies to the specific needs of urban settings is essential for reducing the overall TB burden in Malawi.

Malawi is on track to achieve END-TB strategy goal of 90% TB TSR. This means that provided people access TB services, the outcomes of treatment are excellent for most people with uncomplicated drug-susceptible TB. Children of all ages have excellent treatment outcomes, while HIV-positive adults have poorer treatment outcomes compared with all adults, particularly those receiving treatment in secondary- and tertiary-level facilities. This is likely because the most complicated cases with the poorest prognoses are referred to the tertiary facilities.^37,38^ Despite this, the overall TSR even at tertiary level remains above 80%.

This study has limitations. The analysis was conducted using routine data, which presents issues with data quality and timeliness of reporting. This has been mitigated in view of the fact that the analysis was conducted after ample time had elapsed after the outlined reporting period (quarter 4 of 2022). Our analysis was limited to the reporting format of the facilities, in line with the NTLEP supervision tool. As such, further disaggregation by age and gender is not possible, especially for the treatment success analysis. Despite this, the study included data from all the districts in Malawi, providing a large sample size that results in precision estimates for the TB performance indicators, thus providing a nationally generalisable overview of the TB programme in Malawi.

## CONCLUSION

This study highlights significant progress in TB control in Malawi, evidenced by the overall declining trends in TB incidence and CNRs over the past two decades. The recent stagnation in CNRs, coupled with the persistent gap between estimated TB incidence and notifications, underscores the need for intensified efforts to address undiagnosed and untreated TB cases. There remains a focused epidemic in the urban districts such as Lilongwe and Blantyre. Expanding diagnostic and treatment services, along with targeted interventions such as ACF and community-based awareness campaigns, will be crucial to bridging the case detection gap. Addressing these issues is critical to achieving further reductions in TB incidence and sustaining the gains made in TB control in Malawi.

## Supplementary Material


