# Supplementary material for: Towards TB elimination in Malawi: a 5-year analysis of key indicators for TB control using surveillance data
Source: IJTLD Open. 2025 Oct 10;2(10):590–6. doi: 10.5588/ijtldopen.25.0316 (PMC12517256; doi:10.5588/ijtldopen.25.0316)
Supplement: Supplementary file 1 [file ijtldopen25-0316_supplementarydata1.pdf]

Supplementary materials

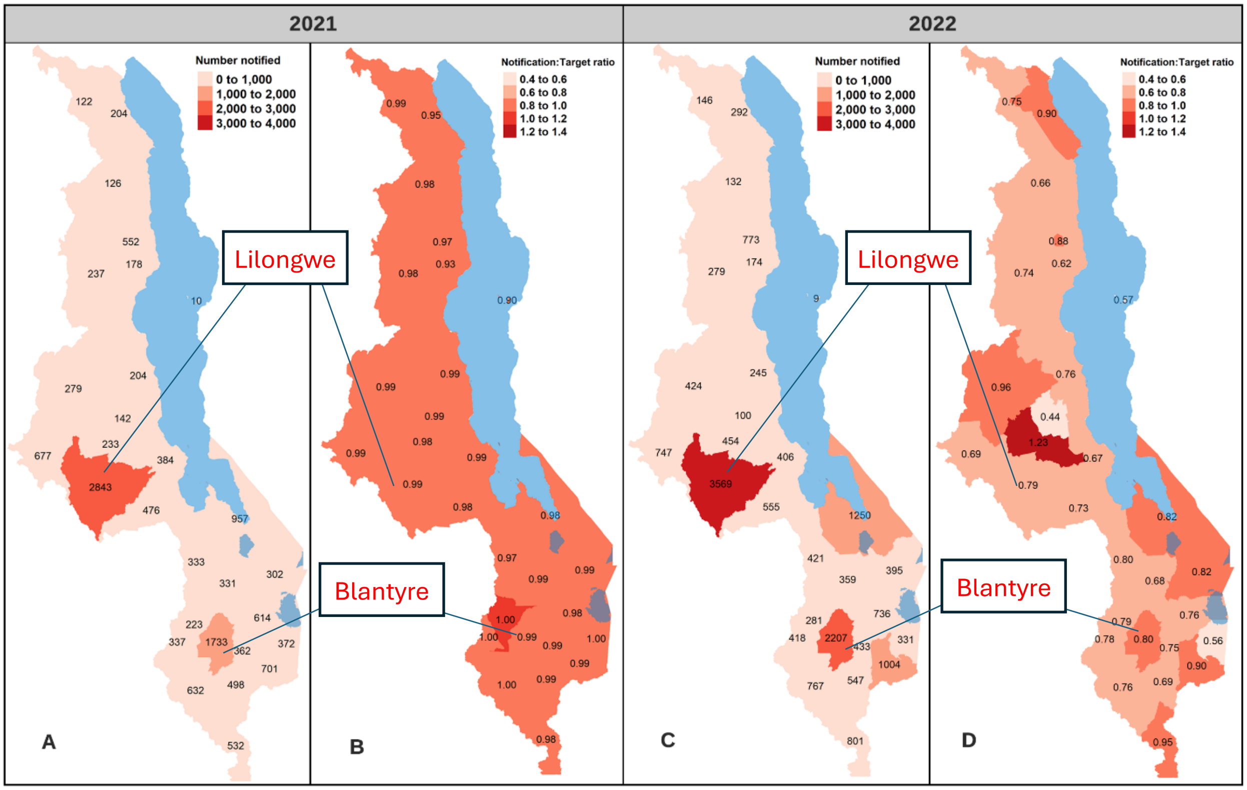

Supplementary figure 1. Subnational tuberculosis (TB) case notifications compared to case detection targets

(A) Subnational TB case notifications in 2021 (B) Notification-to-target ratio for 2021 (C) Subnational TB case notifications in 2022 (D) Notification-to-target ratio for 2022

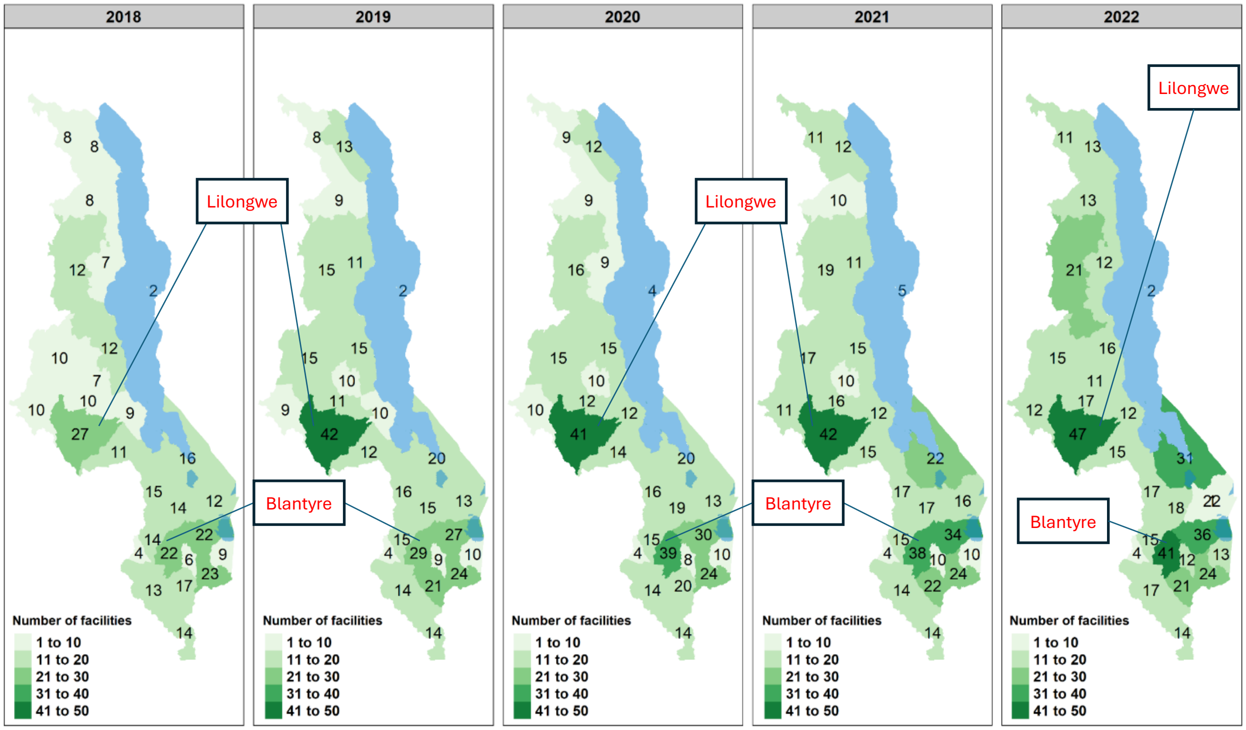

Supplementary figure 2. Distribution of tuberculosis treatment units by district from 2018 to 2022

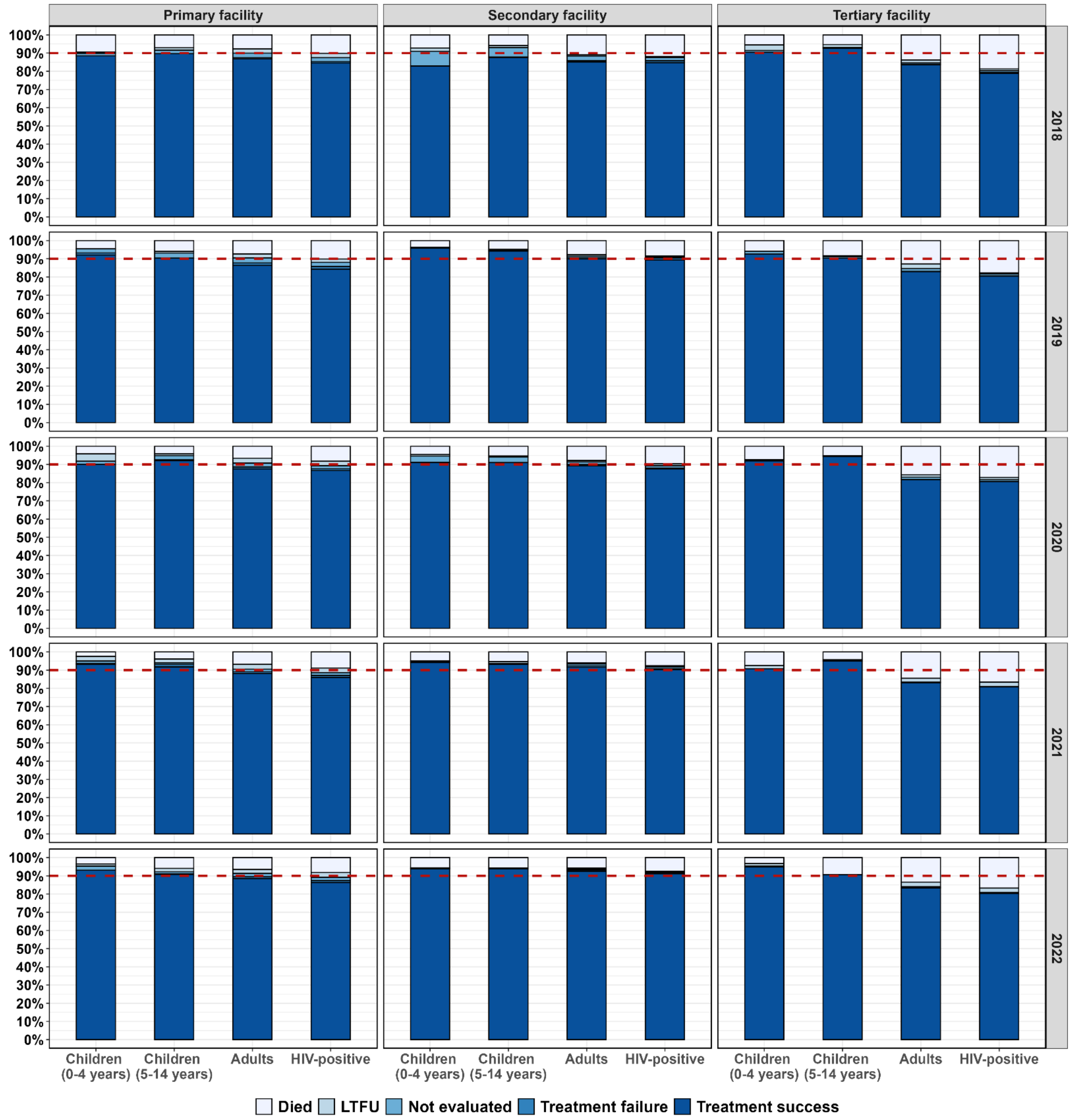

Supplementary figure 3. Disaggregated tuberculosis (TB) treatment outcomes by facility type and reporting year from 2018 to 2022. (The dashed, red line represents the END TB Strategy target for treatment success rate (TSR), which is 90%).

LTFU = loss to follow up
